# Optimizing effector functions of monoclonal antibodies via tailored N-glycan engineering using a dual landing pad CHO targeted integration platform

**DOI:** 10.1038/s41598-023-42925-1

**Published:** 2023-09-20

**Authors:** Ngan T. B. Nguyen, Hau Wan Leung, Kuin Tian Pang, Shi Jie Tay, Ian Walsh, Andre B. H. Choo, Yuansheng Yang

**Affiliations:** Bioprocessing Technology Institute (BTI), Agency for Science, Technology and Research (A*STAR), 20 Biopolis Way, #06-01 Centros, Singapore, 138668 Singapore

**Keywords:** Biotechnology, Cell biology

## Abstract

Monoclonal antibodies (mAbs) eliminate cancer cells via various effector mechanisms including antibody-dependent cell-mediated cytotoxicity (ADCC) and complement-dependent cytotoxicity (CDC), which are influenced by the N-glycan structures on the Fc region of mAbs. Manipulating these glycan structures on mAbs allows for optimization of therapeutic benefits associated with effector functions. Traditional approaches such as gene deletion or overexpression often lead to only all-or-nothing changes in gene expression and fail to modulate the expression of multiple genes at defined ratios and levels. In this work, we have developed a CHO cell engineering platform enabling modulation of multiple gene expression to tailor the N-glycan profiles of mAbs for enhanced effector functions. Our platform involves a CHO targeted integration platform with two independent landing pads, allowing expression of multiple genes at two pre-determined genomic sites. By combining with internal ribosome entry site (IRES)-based polycistronic vectors, we simultaneously modulated the expression of α-mannosidase II (MANII) and chimeric β-1,4-*N*-acetylglucosaminyl-transferase III (cGNTIII) genes in CHO cells. This strategy enabled the production of mAbs carrying N-glycans with various levels of bisecting and non-fucosylated structures. Importantly, these engineered mAbs exhibited different degrees of effector cell activation and CDC, facilitating the identification of mAbs with optimal effector functions. This platform was demonstrated as a powerful tool for producing antibody therapeutics with tailored effector functions via precise engineering of N-glycan profiles. It holds promise for advancing the field of metabolic engineering in mammalian cells.

## Introduction

Monoclonal antibodies (mAbs) have emerged as highly effective cancer therapeutics, owing to their ability to selectively target specific antigens and engage various mechanisms to eliminate cancer cells. In addition to inducing cell death or blocking survival pathways, the Fc region of mAbs plays a pivotal role in mediating innate immune effector mechanisms that are essential for effective cancer therapies^1–3^. These mechanisms include antibody-dependent cell-mediated cytotoxicity (ADCC) and complement-dependent cytotoxicity (CDC), both of which are highly dependent on the N-glycan structures attached to the Fc regions of mAbs^4,5^. Antibodies with non-fucosylated glycans have been shown to exhibit ADCC potency up to 100-fold higher than those fucosylated ones^6,7^. The presence of increased bisecting N-glycans on mAbs has also demonstrated increased ADCC potency but to a lesser extent^8–10^. Galactosylation, on the other hand, did not significantly influence ADCC but played a beneficial role in CDC activation by promoting antibody binding to C1q complexes^11–13^. In contrast, sialylation seemed to reduce CDC and ADCC functions of mAbs, albeit with a less pronounced impact in fucose-free antibodies^14,15^. Given the substantial influence of N-glycans on effector functions, controlling glycosylation profiles of mAbs is crucial in maximizing the therapeutic benefits associated with different effector functions.

With rising interest in improving the biological activities of mAbs, researchers have developed various strategies to engineer glycosylation capacities in mammalian cells, such as Chinese Hamster Ovary (CHO) cells. Engineering host cell genetics has demonstrated remarkable efficiency in modulating mAb N-glycosylation in mammalian cells^16,17^. Gene deletion or RNA interference targeting α-1,6-fucosyltransferase (FUT8) or GDP-fucose transporter (GFT) in CHO cells has resulted in high yield of mAbs with fucose-free glycans, hence improving their ADCC functions^7,18^. Similarly, overexpressing the β-1,4-*N*-acetylglucoseaminyltransferase III (GNTIII) gene in CHO cells has enriched mAbs with non-fucosylated glycans^10^. Interestingly, the study also observed that excessively high GNTIII expression had a diminishing effect on ADCC functions, suggesting the presence of an optimal range of GNTIII expression levels for maximal ADCC improvement. In a subsequent study, higher levels of bisected non-fucosylated antibodies were achieved by overexpressing chimeric GNTIII (cGNTIII), a fusion protein incorporating an α-mannosidase II (MANII) enzyme’s localization signal attached to its own catalytic domain^9^. While this approach produced antibodies with improved ADCC, it also significantly impaired the CDC function. Further studies revealed that co-expressing cGNTIII and MANII genes in CHO cells enabled the production of IgG1 antibodies with enhanced ADCC without compromising CDC activity^9,19^. Collectively, these findings demonstrated feasibility of producing mAbs with customized combinations of different effector functions by modulating the expression levels of glycan-modifying enzymes. However, traditional glycoengineering approaches are incapable of simultaneously modulating the expression levels of multiple genes, as gene deletion or overexpression often results in only all-or-nothing changes without intermediate expression levels.

To achieve precise control over the expression levels of multiple genes, enabling technologies are required to support stable and predictable expression of a large numbers of transgenes. Traditional methods rely on classical transfection of mammalian cells with plasmids, followed by the selection of stable transfectants using antibiotics or metabolic enzyme inhibitors. However, due to the random integration and variation in transgene copy numbers^20,21^, it is nearly impossible to co-express multiple transgenes at defined levels or modulate their expression levels using this random integration approach. In contrast, targeted integration, which can be achieved through recombinase-mediated cassette exchange (RMCE), has emerged as a reliable tool for cell engineering by overcoming position effects and minimize the clonal variations in stably transfected pools^22,23^. RMCE utilizes site-specific recombinases such as Cre, Flp and Bxb1 to consistently insert an expression vector into a predefined genomic locus, referred to as a landing pad, allowing for predictable transgene expression. Although RMCE theoretically enables the insertion of plasmids carrying multiple expression units into a single-landing pad, practically it is very difficult to achieve desirable outcome due to low integration efficiency of the large size plasmids^24^. Consequently, researchers have developed master cells with multiple landing pads to facilitate reliable expression of many transgenes and enable large-scale, sophisticated cell engineering^25,26^. In our own investigations, we found out that up to three transgenes could be consistently inserted and expressed from a single-landing pad in CHO cells by using internal ribosome entry site (IRES)-mediated polycistronic vectors (unpublished data). Beyond integration sites, the DNA regulatory elements also play a role in modulating gene expression levels. Both multiple promoters (MPs) and IRES provide a degree of control over gene expression when co-expressing multiple genes from a single plasmid. While altering promoter strengths can modulate gene expression levels, incorporating MP into a single plasmid often leads to transcriptional interference, where a strong transcriptional unit suppresses the expression of neighboring units^27^. As a result, modulating expression of one gene may have unpredictable effects on the expression of other genes. However, the use of IRES provides an advantage by enabling expression of multiple genes in one transcript without transcriptional interference^28,29^. In addition, the translational level of each gene can be independently adjusted by utilizing IRES mutants with varied strengths^29,30^. Combining the technology of multiple landing pads for targeted integration with IRES elements of different strengths holds the potential to enable precise control over the expression level of multiple genes.

In this work, we aimed to develop a CHO expression platform that enables precise and simultaneous modulation of multiple transgenes’ expression levels, allowing us to design custom N-glycan profiles for mAbs with unique combinations of biological activities. To achieve this, we built two independent landing pads into the CHO targeted integration system, enhancing the capacity and flexibility for multiplexed cell engineering. Subsequently, we combined this platform with an IRES-mediated polycistronic vector system to engineer the expression of MANII and cGNTIII genes in CHO cells, specifically focusing on the production of rituximab IgG1 (anti-CD20-IgG1 antibody) with diverse Fc-mediated effector functions. Rituximab, an extensively used therapy for non-Hodgkin’s lymphoma, possesses the ability to eliminate malignant cells through various mechanisms, including cell death, CDC and ADCC by engaging Fc gamma receptors^31^. By using different strengths of IRES mutants^30^, we were able to independently modulate the expression levels of each gene. We hypothesized that the manipulation of cGNTIII and MANII enzymatic levels would result in distinct glycan profiles on the recombinant anti-CD20 IgG1, ultimately leading to specific combinations of ADCC and CDC activities.

## Results

### Design and characterization of a dual landing pad CHO master cell line for double RMCE

**Figure 1:**
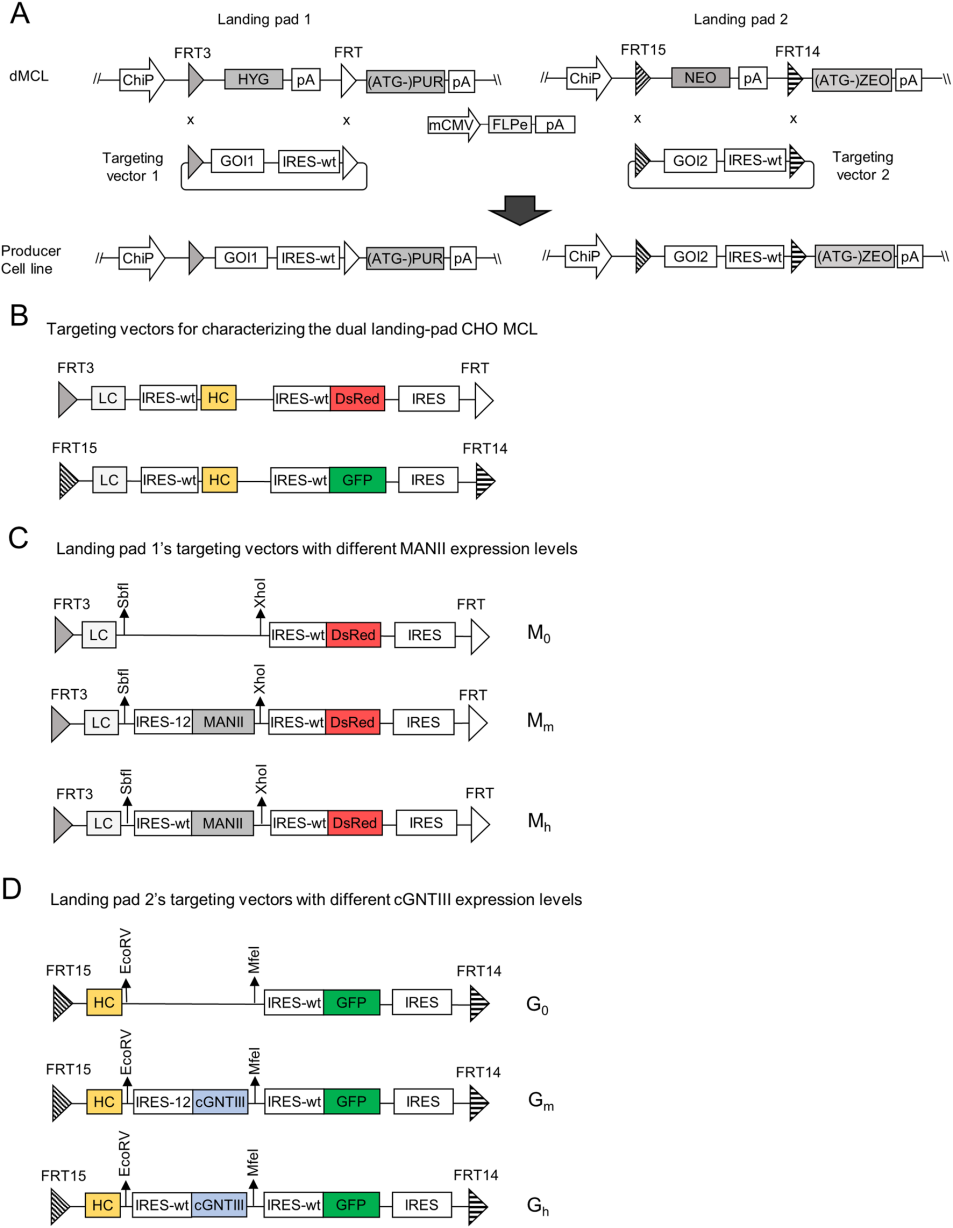
Overview of dual recombinase-mediated-cassette-exchange (RMCE) and targeting vector design for modulating the expression levels of cGNTIII and MANII genes. (**A**) Schematic diagram of dual landing pads CHO master cell line (dMCL) carrying two independent landing pads. The two landing pads express HYG and NEO antibiotic-resistant genes flanked by FRT-FRT3 and FRT15-FRT14, respectively. RMCE activation will replace HYG and NEO with corresponding targeting vectors expressing gene-of-interest (GOI), and initiate expression of PUR and ZEO resistant genes. (**B**) Schematic representation of targeting vectors used to examine the expression capabilities of each landing pad. Each plasmid carried Rituximab LC, HC genes, and DsRed or GFP genes linked through multiple wild-type IRES. (**C**) Schematic representation of targeting vectors specific for landing pad 1 expressing rituximab light chain (LC), α-Mannosidase II (MANII) under different IRES elements to control its translation level. (**D**) Schematic representation of targeting vectors specific for landing pad 2 expressing rituximab heavy chain (HC), chimeric β-1,4-*N*-acetylglucoseaminyltransferase III (cGNTIII) driven by different IRES mutants with vary strength. cGNTIII is a chimeric protein carrying α-mannosidase II (MANII) enzyme’s localization signal fused to its own catalytic domain.

To enable modulation of MANII and cGNTIII gene expression levels in CHO cell lines, we generated a dual landing pads CHO master cell line (dMCL) carrying two independent landing pads with balanced transcription and translation levels (Fig. 1A). The dMCL was constructed by stably integrating a second landing pad vector expressing the neomycin-resistance (NEO) gene into our existing CHO K1 master cell line (MCL)^32^, which already carried a single integrant of landing pad 1 expressing hygromycin-resistance (HYG) gene. In both landing pads, transcription was driven by the chimeric promoter (ChiP) promoter and terminated by the SV40 polyadenylation signal (pA) located upstream of FRT and FRT14, respectively. Consequently, the impaired puromycin resistant gene ((ATG-)Puro) and zeocin resistant regen ((ATG-)Zeo), lacking a start codon located downstream of FRT and FRT14, respectively, remain unexpressed until the activation of RMCE. The specificity of the RMCE reaction targeting each landing pad was conferred by two unique sets of two heterospecific recognition sites: FRT3xFRT for landing pad 1 and FRT15xFRT14 for landing pad 2. These mutant FRTs facilitated multiplexed cassette exchanges through self-recognition capabilities, with minimal cross-interaction, thus ensuring specificity for each landing pad^33^. Each RMCE reaction involved co-transfection of a landing-pad specific targeting vector and an enhanced flippase recombinase (FLPe) expressing vector, followed by appropriate antibiotic selection to generate stably transfected pools. The targeting vectors were designed without promoters and polyadenylation signals to ensure that only cells with correct RMCE survived the antibiotic selection and faithfully expressed all genes carried in the targeting vectors. As a result, the stably transfected cell pools, having the same integration sites, were ideally isogenic with minimizing position effect and gene copy number variations^23,34^.

**Figure 2:**
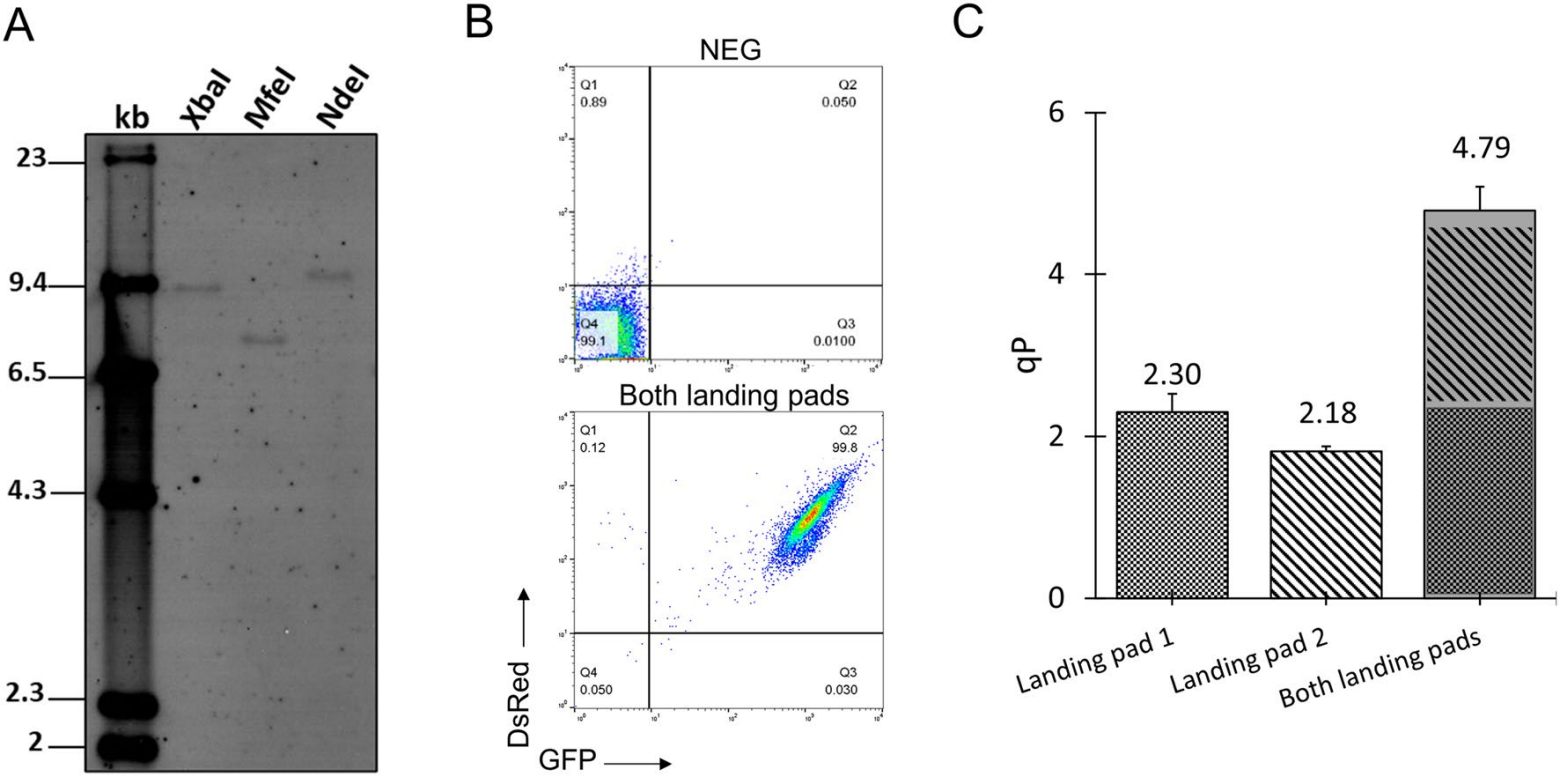
Characterization of the dual landing pads-master cell (dMCL). (**A**) Southern blot analysis of the number of integration sites of the landing pad 2 in dMCL. Genomic DNA of dMCL was digested with either XbaI, MfeI or NdeI, and resolved in 0.7% agarose gel. Membrane was hybridized with DIG-dUTP labeled probes targeting the NEO gene. One DNA band detected in each lane indicates single integration site of the landing pad 2. The expression cassette in landing pad 2 contained only one MfeI restriction site and none of XbaI and NdeI restriction sites. The dMCL were transfected with one or both of targeting vectors as shown in Fig. 1B to characterize expression capacities of each landing pad. (**B**) The stable cell pools generated by transfecting both targeting vectors were subjected to flow cytometry analysis. (**C**) Specific productivity (qP) of producer cell lines expressing mAbs from single landing pads and both landing pads during seven-day fed batch culture.

The landing pad 1 in the dMCL had previously been confirmed to be a single integrant in the parental MCL in our previous study^32^. We further examined the number of integration sites of the landing pad 2 in the dMCL via Southern blot. The dMCL exhibited a unique single DNA band upon digestion with XbaI, MfeI or NdeI restriction enzymes (Fig. 2A), indicating that it carried a single integrant of the landing pad 2 vector. To assess the expression capability of each landing pad, we performed RMCE to replace the HYG or/ and NEO gene with rituximab IgG1 mAb expression cassettes to generate single plasmid and double plasmids integrated stable cell pools. The targeting vector for landing pad 1 was designed to carry rituximab light chain (LC), heavy chain (HC) and DsRed genes linked through multiple wild-type Encephalomyocarditis virus (EMCV) IRES (IRES-wt) (Fig. 1B). Similarly, each targeting vector for landing pad 2 contained EMCV IRES-linked rituximab LC, HC and GFP genes. An additional wild-type EMCV IRES was placed downstream of DsRed or GFP gene to activate the expression of (ATG-)Puro and (ATG-)Zeo genes, respectively, after RMCE. The inclusion of DsRed and GFP genes in each targeting vector allowed for quick and reliable analysis of expression homogeneity in stably transfected cell populations using flow cytometry. Single plasmid-integrated stable cell pools expressed the mAb from either of the landing pads, while double plasmid-integrated stable cell pools expressed the mAb from both landing pads. The double stable cell pools exhibited homogenous expression of both DsRed and GFP genes, indicating that the isolation of single cell clone to evaluate the impact of overexpressing transgenes on phenotype could be avoided (Fig. 2B). Subsequently, these stable cell pools were evaluated for specific antibody productivity (qP) in 7-day fed-batch cultures. The two landing pads displayed comparable expression levels when a single plasmid was integrated. When two plasmids were integrated, the total expression levels from the two landing pads were additive (Fig. 2C). This demonstrated that the two loci expressed independently with minimal interference, ensuring the reproducibility of each landing pad’s predetermined expression level. These findings established the dMCL as an ideal platform for modulating the expression of multiple genes.

### Generation of stable mAb-producing CHO pools expressing various levels of MANII and cGNTIII genes

To study the impact of different enzyme combinations on the N-glycan profile of rituximab and its effector functions, we generated stable mAb-producing CHO cell pools expressing various levels of MANII and cGNTIII. These stable mAb-producing cell pools were generated through two rounds of sequential RMCE. In the first round, targeting vectors carrying LC and MANII genes were integrated into landing pad 1 (Fig. 1C). This was followed by the integration of targeting vectors carrying HC gene and cGNTIII gene into landing pad 2 (Fig. 1D). The expression levels of MANII and cGNTIII were modulated at three levels: zero (no expression), medium, and high, achieved by using different IRES upstream of the corresponding (Fig. 1C, D). High gene expression level was driven by the wild-type IRES (IRES-wt), while medium expression was controlled by an IRES mutant (IRES-12) which was previously demonstrated to achieve approximately 15% translational strength of IRES-wt^30^. To indicate the expression level of a gene, a subscript was added to the gene name abbreviation. M_0_, M_m_ and M_h_ represented zero, medium and high expression levels of MANII, respectively. Similarly, G_0_, G_m_ and G_h_ denoted the expression levels of cGNTIII as zero, medium, and high, respectively. In total, nine different conditions with varying MANII and cGNTIII expression levels were generated in the stable cell pools.

**Figure 3:**
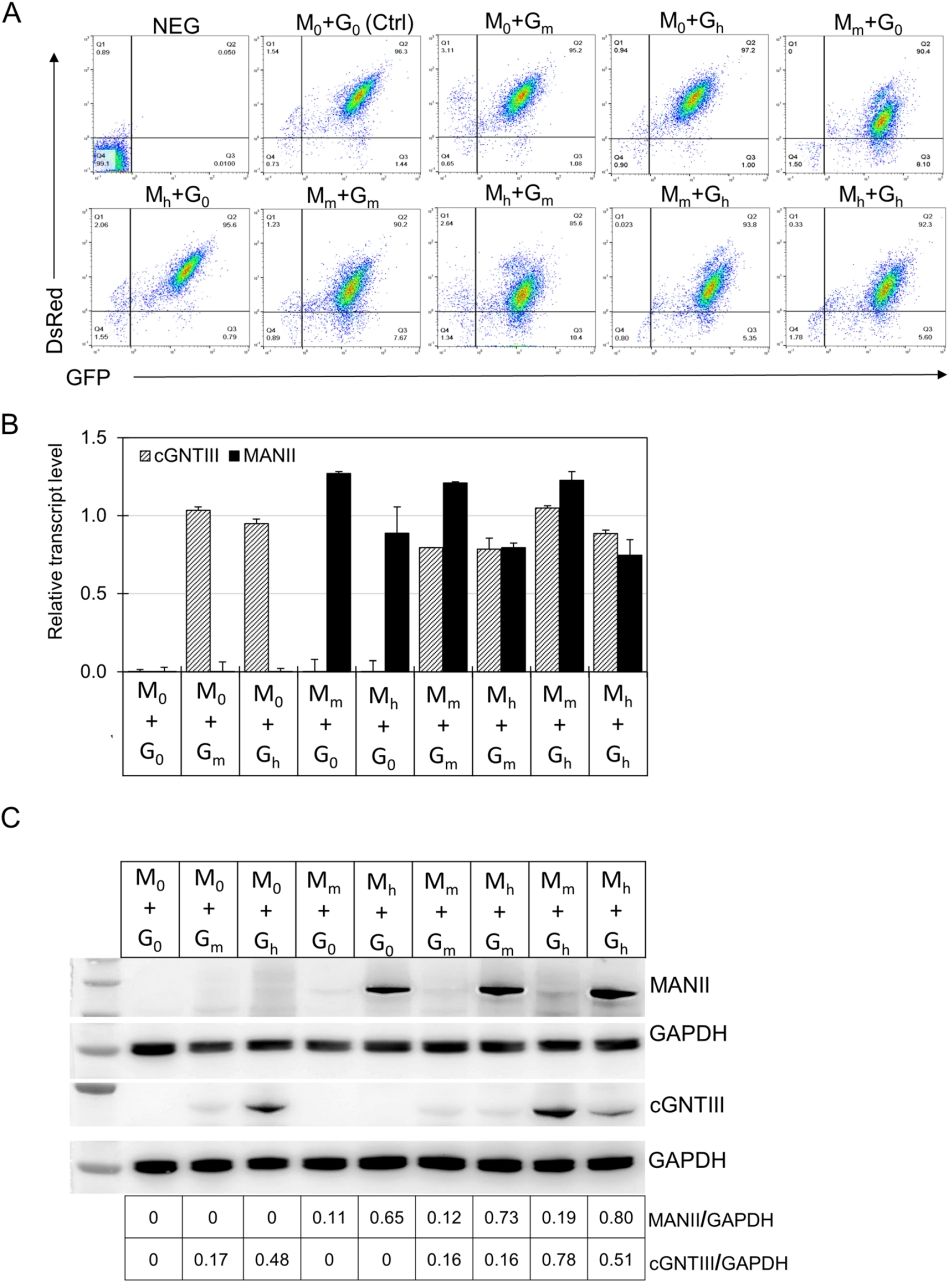
Characterization of mAb-producing stable CHO pools expressing different levels of MANII and cGNTIII genes. The expression of each gene was varied in three levels: zero (no expression, M_0_ and G_0_), medium (M_m_ and G_m_) and high (M_h_ and G_h_), resulting in nine combinations of different expression levels of MANII and cGNTIII genes. Two stable pools were generated independently through RMCE for each combination of MANII and cGNTIII expression levels. The NEG pools were dMCL without RMCE. Control pools expressed Rituximab LC and HC only. (**A**) Flow cytometry analysis of the nine stable mAb-producing cell pools after they were fully recovered from antibiotic selection. Representative data from one replicate pool of each condition was showed. (**B**) Relative MANII and cGNTIII transcript levels in the stable pools was analysed by quantitative real-time PCR (qRT-PCR). β-actin gene was used as internal control. Each point represents the average and standard deviation of relative transcripts from two independent stably transfected pools. (**C**) Western blot analysis of MANII and cGNTIII protein levels in the stable pools. GAPDH was used as loading control. Uncropped blot images are available in Supplementary Fig. S1. The relative expression of MANII and cGNTIII genes was normalized to the corresponding loading control in each sample.

First, we evaluated the gene expression homogeneity of the stable cell pools using flow cytometry. Compared to the negative control, more than 90% of the cell population in all pools exhibited double positivity for both DsRed and GFP (Fig. 3A), indicating a robust and uniform expression profile from both landing pads. This demonstrated the suitability of these stable pools for producing different mAb variants without the need for clone isolation. Subsequently, we performed RT-PCR analysis of mRNA levels to confirm the successful overexpression of both MANII and cGNTIII in the stably transfected pools (Fig. 3B). Furthermore, western blot analysis was conducted to examine the expression levels of MANII and cGNTIII genes controlled by different IRES elements (Fig. 3C). The relative abundance of each enzyme in the samples was quantified by normalizing the band intensity to the corresponding GAPDH protein. The protein bands corresponding to MANII and cGNTIII were most pronounced in the stable pools expressing MANII and cGNTIII driven by the IRES-wt. Their relative abundance levels ranged from 0.65 to 0.80 for MANII and 0.48–0.78 for cGNTIII. In contrast, the application of IRES-12 on either MANII or cGNTIII effectively reduced their relative expression levels to 0.11–0.19 and 0.16–0.17, respectively (Fig. 3C). Since the relative transcript levels of both MANII and cGNTIII genes were similar in all stable pools (Fig. 3B), the observed variations in protein levels, as detected in the western blot analysis, resulted from the different translational strengths of different IRES elements rather than intrinsic differences in expression capacities of the two landing pads. This further validated the ability to modulate the expression levels of transgenes using appropriate IRES elements.

### Production of rituximab with various N-glycan profiles by using CHO cells that co-express cGNTIII and MANII at different protein levels

**Figure 4:**
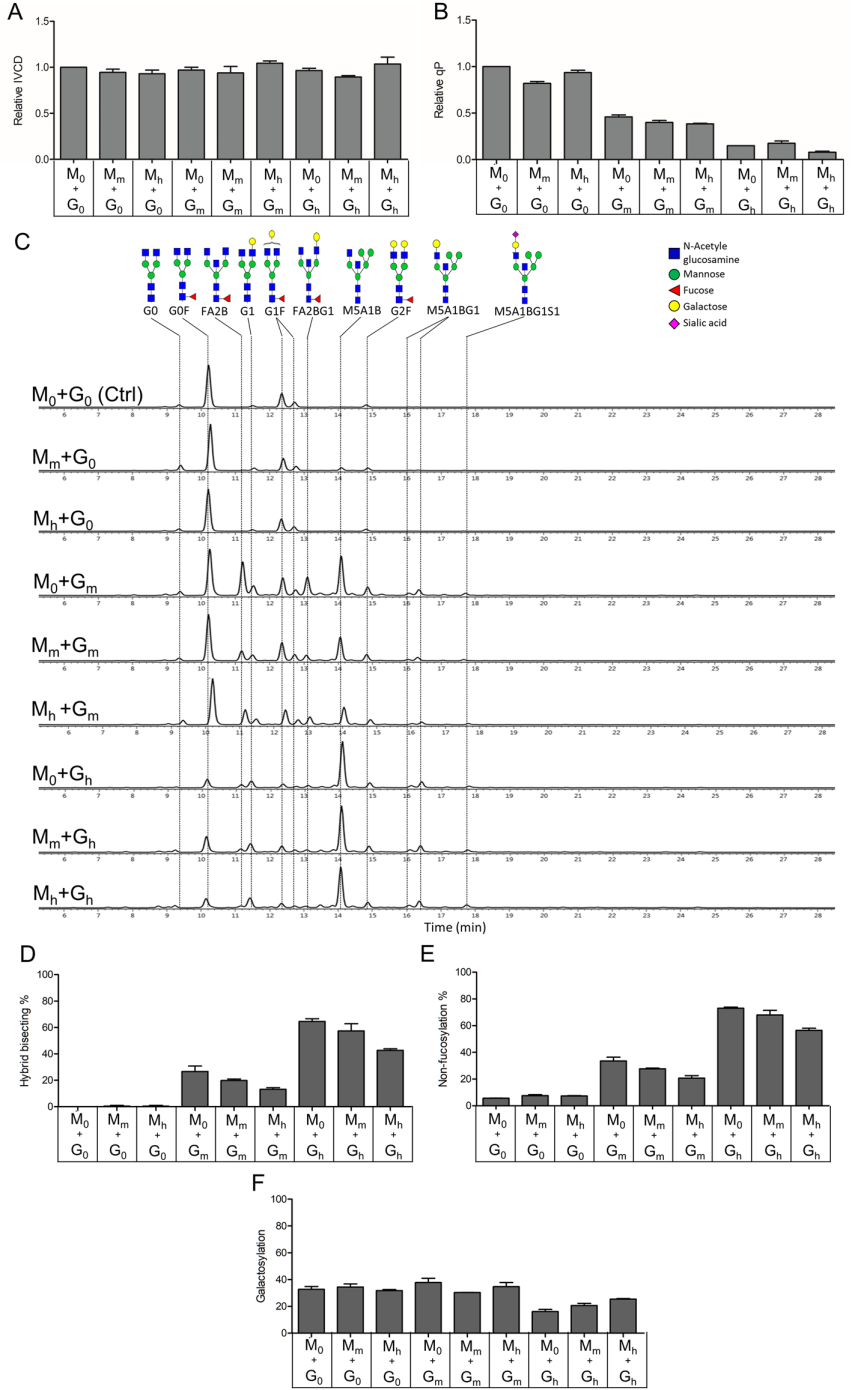
Impact of varying expression of MANII and cGNTIII genes on Rituximab production and N-glycosylation levels. All stable pools expressing mAbs and different levels of MANII and cGNTIII were subjected to seven-day fed-batch production. (**A**, **B**) The integrated viable cell density (IVCD) and specific productivity (qP) of each stable pool were represented as relative change to the control pool. (**C**) N-glycan profiles of Rituximab produced by combination of different MANII and cGNTIII expression levels were presented in aligned HILIC chromatograms from one replicate stable pool of each condition. (**D**–**F**) Relative levels of hybrid bisecting glycans, non-fucosylation and galactosylation of glycoengineered IgG1 produced by combination of different MANII and cGNTIII expression levels.

The mAb-producing stable pools were characterized for cellular growth, antibody productivity in 7-day fed-batch production, followed by N-glycosylation analysis of the purified antibodies. Supplementary feed was added and culture supernatants were harvested at exponential growth phase to minimize the potential effects of nutrient depletions. The different combinations of MANII and cGNTIII gene expressions did not impact the cell growth in any of the nine stable cell pools, as indicated by the unchanged integrated viable cell density (IVCD) compared to the control culture (Fig. 4A). Overexpressing MANII gene alone caused little change in the specific antibody productivity (qP) compared to the control. However, the productivity was significantly affected in the stable pools expressing the cGNTIII gene alone or in combination with MANII (Fig. 4B). The qP was decreased by half when cGNTIII gene was expressed at medium levels, and high expression of cGNTIII gene further reduced the antibody productivity to approximately 0.2-fold of the control culture. This finding was consistent with a previous study where high-level of GNTIII gene expression showed adverse effects on antibody production yield^10^.

The N-glycosylation profiles of mAbs produced in stable pools with different levels of MANII and cGNTIII were analyzed using hydrophilic interaction liquid chromatography (HILIC). Previous studies have consistently reported that mAbs produced in the wild-type CHO cells are predominantly fucosylated with minimal galactosylation and terminal sialylation^32,35^. Overexpressing cGNTIII in mammalian cells has been shown to result in antibodies with bisecting glycan structures and reduced fucose content^9,10^. Consistent with these studies, our analysis revealed that the predominant glycan form in the control culture was G0F, a fucosylated species, with minimal detectable bisecting GlcNAc structures (Fig. 4C). Overexpressing MANII alone, regardless of its expression level, produced antibodies with N-glycan profiles similar to those of the control pools. In contrast, overexpression of cGNTIII gene led to the production of many bisecting glycans such as FA2B, FA1BG1, M5A1B, and M5A1BG1, while many of these glycan structures lacked fucose residues (Fig. 4C). Pools expressing high levels of cGNTIII predominantly produced IgGs with M5A1B, a hybrid bisecting non-fucosylated structure, whereas medium expression of cGNTIII produced lower levels of M5A1B glycans and moderate levels of bisecting fucosylated glycans like FA2B, FA2BG1 (Fig. 4C). Quantitatively, high-expressing cGNTIII pools produced 40–60% of IgG1 with hybrid bisecting glycans (M5A1B), while medium-expressing cGNTIII pools produced 10–20% IgGs carrying M5A1B glycans (Fig. 4D). As these hybrid structures lacked fucose residues, the non-fucosylation levels in high-expressing cGNTIII pools increased to nearly 70% compared to 5% in the control pools, whereas medium-level cGNTIII expression resulted in lesser non-fucosylated IgGs, ranging from 20 to 30% (Fig. 4E). Additionally, high-level overexpression of cGNTIII led to a reduction in the galactosylation level of IgG1s (Fig. 4F) due to the shift from FA2BG1 and FA2B toward M5A1B glycans (Fig. 4C). Conversely, medium-level overexpression of cGNTIII did not impair the galactosylation of IgG1s compared to control pools. While MANII overexpression alone did not significantly impact IgG glycan profiles, its co-expression with cGNTIII suppressed the effect of cGNTIII on bisecting glycan, non-fucosylation, and galactosylation in a dose-dependent manner (Fig. 4C–E). In pools expressing mid-level cGNTIII, the peaks corresponding to bisecting M5A1B, FA2BG1 and FA2B gradually reduced as MANII expression increased (Fig. 4C). The abundance of hybrid bisecting glycans was highest in pools expressing cGNTIII alone, and their levels gradually decreased with increased expression of the MANII gene (Fig. 4D). Among the high-expressing cGNTIII pools, the highest non-fucosylation induction (72%) was observed in the absence of MANII, followed by medium- and high-levels of MANII expression with 68% and 56% non-fucosylation, respectively (Fig. 4E). A similar trend was also observed in pools expressing a medium level of cGNTIII in conjunction with varying levels of MANII gene expression. Furthermore, the reduced galactosylation levels induced by high-level overexpression of cGNTIII gene was reversed by increasing MANII levels in a dose-dependent manner (Fig. 4F).

### The impact of different N-glycosylation profiles on rituximab effector functions

**Figure 5:**
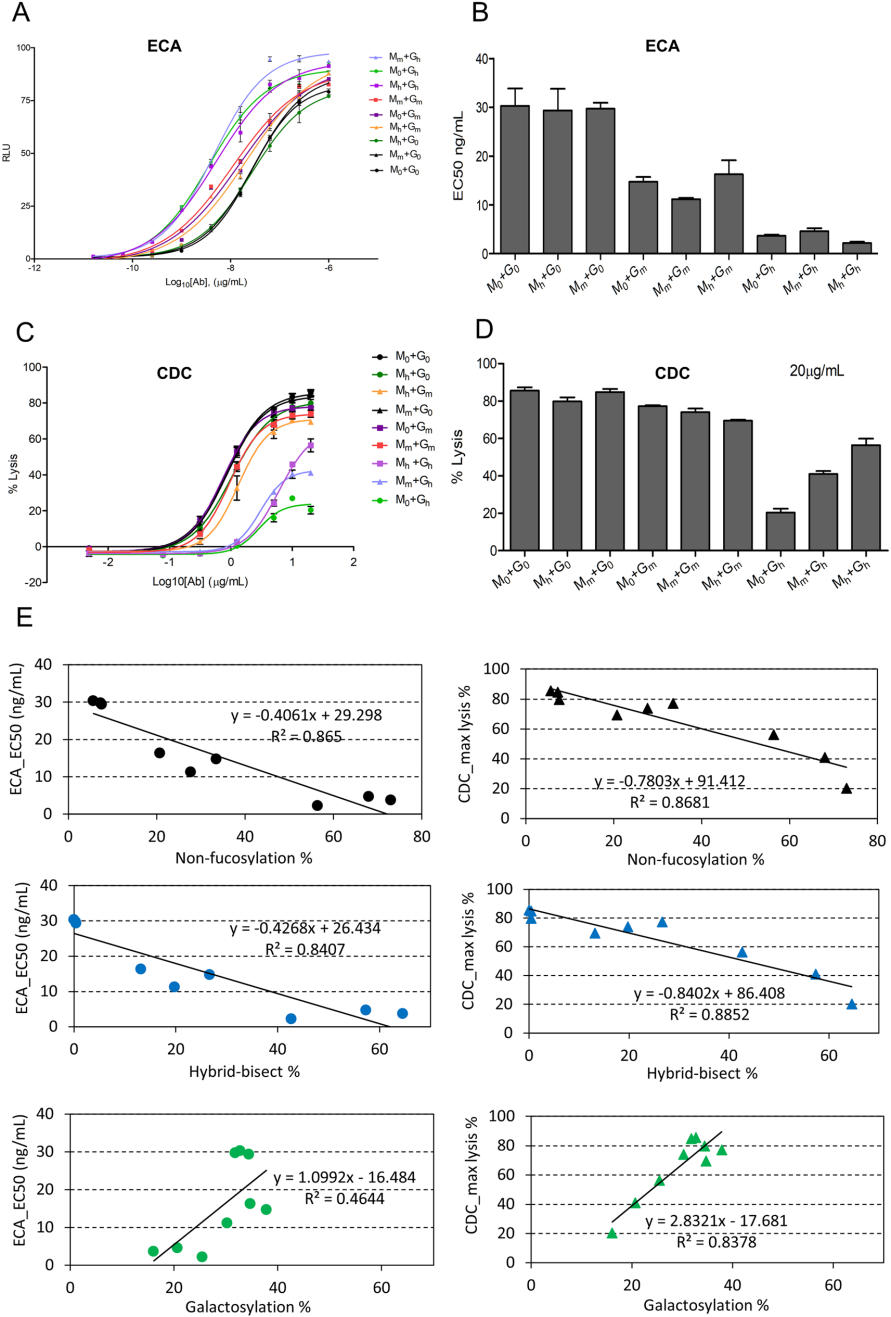
Analysis of Fc-mediated effector functions of modified Rituximab produced by stable cell pools expressing various levels of MANII and cGNTIII. ECA analysis using Jurkat/NFAT-*luc* (FcγRIIIa) as effectors and human WIL2-S cells as targets. (**A**) Dose–response curves of ECA induced by various variant Rituximab. Data points indicate mean values of luciferase signal ± SD, n = 4. Data was fitted to a 4PL curve using GraphPad Prism software. (**B**) EC50 values were calculated for each variant Rituximab. Bars representing mean values of EC50 ± SD, n = 4. (**C**, **D**) CDC analysis using Raji cells in the presence of human serum as a source of complement. (**C**) Dose–response curves of CDC against Raji cells. Data points indicate mean values of specific lysis ± SD, n = 4. Data was fitted to a 4PL curve using GraphPad Prism software. (**D**) Lysis % at antibody concentration of 20 µg/mL. Bars represent mean values of specific lysis ± SD, n = 4. (**E**) Correlation plots (N-glycosylation outcome vs. effector function) and the correlation coefficient (R). EC50 value representing ECA activities of mAbs were plotted against the levels of non-fucosylation %, hybrid-bisect %, and galactosylation % (left panels). Max lysis % representing CDC activities of mAbs were plotted against the levels of non-fucosylation %, hybrid-bisect %, and galactosylation% (right panels). EC50 value, max lysis %, non-fucosylation %, hybrid-bisect %, and galactosylation % were the mean values obtained from two independent stable cell pools.

In the next step, we examined the relationships between the different glycovariant rituximab IgG1s and their Fc-mediated biological functions. The purified antibodies were analyzed for their ability to induce effector cell activation (ECA) against CD20-positive WIL2-S cells (Fig. 5A, B) and CDC against CD20-positive cancer cells RajiB in the presence of human serum (Fig. 5C, D). The levels of ECA induced by different antibody samples were assessed using the ADCC Reporter Bioassay kit. This kit utilizes engineered Jurkat cells that express the FcγRIIIa receptor and contain a nuclear factor of activated T-cells (NFAT) response element driving the expression of firefly luciferase as effector cells. Instead of directly measuring target cell killing, which defines ADCC, the assay kit evaluates the activation of the ADCC mechanism of action pathway. Therefore, we utilized the term ECA instead of ADCC potency as a more accurate measure to reflect the results. A previous study demonstrated a strong correlation between the biological activity determined using this assay kit and the classic peripheral blood mononuclear cells (PBMC)-based ADCC assay^36^. Consequently, the ECA measured by the ADCC Reporter Bioassay kit serves as a reliable indicator of ADCC potency in different antibody samples, providing valuable insights into their efficacy.

Overexpressing MANII gene alone did not affect the ECA function of IgG1, regardless of its expression level (Fig. 5A, B). This result was expected since the mAbs produced in these pools exhibited similar glycan profiles to the control (Fig. 4C). In contrast, moderate-level overexpression of the cGNTIII gene significantly improved ECA potency of IgG1 by two-fold, as indicated by a 50% reduction in EC50 values (Fig. 5B). Rituximabs produced in pools with high-level expression of cGNTIII showed a leftward and upward shift in the ECA dose–response curves (Fig. 5A), indicating improved ECA potency and maximal efficacy. The ECA EC50 value markedly reduced to a range of 2.01–4.30 ng/mL, which were 15- to 7-fold improvement, respectively, compared to the value of 30 ng/mL in unmodified antibodies (Fig. 5B). Notedly, cGNTIII could improve ECA functions of IgG1 independent of different expression levels of MANII gene. Previous studies have reported that antibodies carrying non-fucosylated glycans display significantly higher ADCC potency, up to 100-fold, compared to their fucosylated counterparts^6,7^. The observed lower levels of ECA in our study could potentially be attributed to two factors: either incomplete removal of fucose in our antibody samples or the utilization of different assay methodologies.

While medium or high levels of MANII overexpression alone resulted in little changes to the CDC function of Rituximab IgG1, a slight rightward shift in the CDC dose–response curves was observed with medium expression of cGNTIII, followed by a more pronounced shift with high expression of this enzyme (Fig. 5C). At the saturating dose (20 µg/mL), high-level overexpression of cGNTIII alone drastically reduced the maximal efficacy of Rituximab CDC by four-fold compared to the control samples (Fig. 5D). However, the weakened CDC functions were mitigated by increasing the expression of MANII gene in the high-expressing cGNTIII pools, resulting in only a two-fold and 0.75-fold reduction in the max CDC of IgG1 produced by M_m_ + G_h_ and M_h_ + G_h_ pools, respectively (Fig. 5D). Co-expression of MANII and cGNTIII genes at high levels produced rituximab IgG1 with the highest ECA potency and only approximately a 20% reduction in CDC activity (Fig. 5B, D).

To examine the relationship between glycosylation outcomes and the two Fc-mediated effector functions of IgG1, we plotted the levels of non-fucosylation, hybrid bisect, galactosylation of mAbs produced by CHO pools expressing different levels of MANII and cGNTIII against their corresponding ECA and CDC activities, respectively (Fig. 5E). Previous studies have reported that a high proportion of non-fucosylated IgG1 enhances ADCC functions^9,10,19^. Consistently, we observed a strong correlation between Fc non-fucosylation and ECA efficacy (R^2^ of 0.86), whereby increased non-fucosylation levels resulted in reduced ECA EC50 values (Fig. 5E left panels). As expected, ECA efficacy also strongly correlated with the percentage of hybrid-bisect structures (M5A1B) (R^2^ of 0.84), as a significant proportion of these structures in our samples lacked fucose residues (Fig. 4C), directly contributing to the non-fucosylation outcome. Hybrid structures has been reported to impair CDC activity of mAbs^4,37^. Similarly, we found that CDC maximal efficacy correlated strongly with both hybrid-bisect and non-fucosylation levels (R^2^ of 0.88 and 0.86, respectively) (Fig. 5E right panels). While Fc galacosylation showed only a weak correlation with ECA efficacy (R^2^ of 0.46), this variable exhibited a high correlation with CDC (R^2^ of 0.83). These findings align with previous studies highlighting the role of Fc galactosylation in enhancing CDC activation^12,13^.

## Discussion

There have been many reports on glycoengineering involving the stable expression of single or multiple glycosyltransferases. However, there is a lack of studies on modulating expression levels of multiple genes to obtain desirable glycan structures. In this study, we utilized adjustable IRES-based polycistronic expression cassettes integrated into the CHO genome to achieve stable expression of multiple glycosyltransferases genes at different levels. Our approach involved the generation of a novel RMCE-based targeted integration platform in CHO cells, which comprised two independent landing pads with balanced expression levels. We co-expressed the mAb genes along with two glycosyltransferase genes, cGNTIII and MANII, by integrating polycistronic vectors into the two pre-determined loci in the master cells. By using IRES elements with adjustable translational strength, we were able to simultaneously modulate the expression levels of cGNTIII and MANII, leading to the production of IgG1s with distinct glycan profiles and unique combinations of biological activities. Combining medium levels of cGNTIII overexpression with any levels of MANII overexpression increased non-fucosylation by 20% without altering galactosylation levels in the mAbs. As a result, these modified IgG1s exhibited a two-fold improvement in ECA without compromising their CDC function compared to the unmodified antibodies. Increasing the overexpression levels of the cGNTIII gene alone resulted in up to 70% non-fucosylated mAbs, further enhancing ECA by 15-fold. Nevertheless, their CDC functions were significantly impaired due to the presence of a high proportion of hybrid bisecting glycans with low galacosylation levels. To address this issue and maintain the improvement in ECA activity while mitigating the adverse effect on CDC, we co-expressed high levels of the MANII gene with high levels of the cGNTIII gene. This combination reduced the negative impact on CDC while preserving the enhancement of ECA activity. Nonetheless, we observed a notable reduction in antibody productivities upon co-expressing cGNTIII and ManII, indicating the combination of cGNTIII and ManII is not an ideal choice for engineering glycosylation on therapeutic antibodies. Alternative glycosylation enzymes could be explored for obtaining both high productivity and desirable functionalities. Additionally, enhanced antibody productivities could be achieved by incorporation of multiple copies of LC and HC genes in the targeting vectors, coupled with the development of master clones having landing pads integrated into more active genomic sites (unpublished data).

Our technology, as a constitutive expression system, offers advantages over inducible systems commonly used to modulate gene expressions using small-molecule inducers. For example, Chang and colleagues recently demonstrated concurrent control of Fc galactosylation and fucosylation by using two synthetic gene expression circuits (FUT8, B4GALT1) regulated by doxycycline and abscisic acid^38^. However, the use of small-molecule drugs in scaling up often presents challenges due to potential process inconsistency and increased cost associated with additional purification steps required to remove these compounds. Consequently, optimizing glycosylation levels for each new antibody typically involves initial optimization using small molecule-induced systems before transitioning to suitable constitutive platforms for production. Our technology eliminated the need to switch between various expression systems since both engineering and production requirements can be met within a single platform. In addition, our targeted integration platform, featuring two independent landing pads, offers increased payload capacity for engineering biosynthesis pathways like N-glycosylation. Each locus possesses a well-defined and distinct expression capability, enabling predictable and precise control over gene expression. In combination with the availability of a wide range of IRES mutants with varying translational strength^30^, our platform allows for modulating the stable expression of multiple genes to the appropriate levels, thereby achieving the desired glycosylation patterns tailored to specific biological functions. This targeted integration platform with multiple landing pads also holds great potential for various applications in mammalian synthetic biology and recombinant protein production.

It has been reported that high levels of GNTIII gene overexpression are associated with reduced antibody production^10^, potentially due to the inhibitory effect of excessive glycosyltransferase production on cell growth and overall antibody yield. In our work, we observed dose-dependent decrease in antibody titer with increasing levels of cGNTIII gene overexpression (Fig. 4B). However, contrary to expectations, high levels of cGNTIII enzyme did not suppress cell growth during fed-batch cultures (Fig. 4A). It is important to note that prior work involving transient expression of antibodies often results in vector loss during repeated cell replication cycles^21,39^, which could contribute to the observed reduction in antibody yield during scale-up. By integrating transgenes into the host genome, our system can overcome this issue and allows for stable expression of antibody genes over an extended period. Therefore, our finding suggests that cGNTIII enzymes influence antibody production in CHO cells through mechanisms other than cell growth inhibition. One possible explanation is that cGNTIII overexpression may impair the proper folding of antibodies in the Endoplasmic reticulum (ER) and affect the secretion of nascent proteins. Further investigations are needed to understand the influence of glycosyltransferases on protein folding and secretion, which will contribute to the development of improved strategies for antibody engineering in the future.

Our work provided new insights into the regulatory role of chimeric GNTIII and MANII enzymes in the formation of bisecting and non-fucosylated glycans. Previous research demonstrated that overexpression of chimeric GNTIII alone predominantly directed antibody glycan processing toward hybrid bisecting non-fucosylated structures^9^, resulting in improved ADCC functions but reduced CDC activity. In contrast, co-expression of chimeric GNTIII and MANII generated bisecting non-fucosylated glycans without the hybrid forms, leading to increased ADCC efficacy while preserving CDC activity^9,19^. It was hypothesized that MANII overexpression could redirect the glycan biosynthetic pathway from hybrid to complex structures. Our findings supported this hypothesis and further demonstrated that MANII, when co-expressed with cGNTIII, effectively reduced the abundance of hybrid bisecting N-glycans in a dose-dependent manner (Fig. 4C, D).

## Methods

### Cell culture and media for maintenance of dual landing pad CHO master cell line (dMCL)

The dMCL was generated by transfecting a second landing pad vector into our previously established CHO K1 master cell line (sMCL), which was derived from the parental ATCC CHO K1 cells and already contained the first landing vector^32^. The structures of the first and second landing pad vectors were shown in Fig. 1A. The sequences of the DNA elements in the first landing pad vector were described in our previous study^32^. The second landing pad vector shares the same structure and elements as the first, except that HYG was replaced with NEO, and FRT3xFRT was replaced with FRT15xFRT14. The sequences of the hetero-specific recombination sites FRT15 and FRT14 were described in a previous publication^33^. The sMCL transfected with the second landing pad vector were culture in a protein-free medium (PFM) containing 600 µg/mL G418 (Thermo Fisher Scientific) using 125 mL shake flasks (Corning) placed in a humidified Kuhner shaker (Adolf Kühner AG) with 8% CO_2_ at 37 °C. The PFM was prepared by mixing HyQ PF (GE Healthcare Life Sciences) and CD CHO (Thermo Fisher Scientific) in a 1: 1 ratio, supplemented with 1 g/L sodium carbonate (Sigma), 6 mM glutamine (Sigma), and 0.1% Pluronic F-68 (Thermo Fisher Scientific). Clones were subsequently isolated and screened for single-copy integration of the second landing vector through Southern blotting analysis. The obtained dMCL, containing single copy integration of the two landing pads, was maintained in the PFM containing 600 µg/mL Hygromycin (Thermo Fisher Scientific) and 600 µg/mL G418 in 125 mL shake flasks in the humidified Kuhner shaker. Regular passage of the cells was conducted every 3–4 days by diluting the cultures to a density of 3 × 10^5^ cells/mL in 15 mL of fresh medium. Cell counting was carried out using a Vi-Cell XR viability analyzer and trypan blue exclusion method (Beckman Coulter).

### Construction of targeting plasmid vectors for expressing Rituximab IgG1 and different levels of MANII and cGNTIII genes

The vector expressing improved FLP recombinase (FLPe) was described in our previous study^32^. Two control targeting vectors carrying expression cassettes of FRT3-LC-IRES-DsRed-IRES-FRT and FRT15-HC-IRES-GFP-FRT14 were constructed by GenScript. These vectors included two unique restriction sites, SbfI and XhoI between LC and IRES-wt-DsRed, EcoRV and MfeI between HC and IRES-wt-GFP, for insertion of additional expression units. To construct targeting vectors expressing high and medium levels of the MANII gene, two synthesized units IRES-wt-MANII and IRES-12-MANII, were inserted into the FRT3-FRT control vector using SbfI and XhoI, respectively. Similarly, two synthesized units, IRES-wt-cGNTIII and IRES-12-cGNTIII, were inserted into the FRT15-FRT14 control vector using EcoRV and MfeI, respectively. The sequences of the wild-type IRES, IRES-12, DsRed, GFP, rituximab LC cDNA, and HC cDNA were described in our previous study^32,33^. The human α-mannosidase II (MANII) cDNA sequence (U31520.1) was obtained from GenBank, followed by the addition of a sequence encoding a c-Myc-epitope tag at the C-terminus. The cGNTIII was a chimeric gene consisting of a localization domain from the human MANII enzyme fused to the N-terminus of the catalytic domain of GNTIII, followed by a C-terminal c-Myc-epitope tag^9^.

### Generation and characterization of IgG-producing stable cell pools via sequential RMCE

The stable cell pools co-expressing both IgG1 and different levels of glycosyltransferase genes were generated by performing two rounds of RMCE reactions in the dMCL. In the first round, the dMCL was co-transfected with an appropriate targeting vector for landing pad 1 and a vector expressing FLPe using Amaxa SG Cell Line 4D-Nucleofector X Kit and program FF-137 (Lonza). Stable transfectants were then selected in PFM containing 20 µg/mL of puromycin (InvivoGene) to generate stably transfected pools. The protocols for transfections and generation of stable pools were described in our previous study^32^. The recovered stable cell pools were then subjected to the second round of transfection, where they were co-transfected with a second targeting vector for landing pad 2 and a FLPe expression vector following similar protocols as described above. Stable transfectants were then selected in PFM containing 600 µg/mL of zeocin (InvivoGene). When the viability of stable pools recovered to greater than 90%, the final desired stable cell pools were maintained in the PFM containing both puromycin at 20 µg/mL and zeocin at 600 µg/mL. The expression of DsRed and GFP in each stable pool was analyzed on a BD FACSCalibur, and the data were analyzed using FlowJo™ 10.7.2 software (BD Life Sciences). The growth and productivity of stable pools were characterized in fed-batch cultures as described in our previous study^32^. Two sets of ten million cells were collected from each fed-batch culture at day 7 and saved for later analysis of mRNA and protein levels. The culture supernatant was also harvested at day 7 for later purification and N-glycan analysis.

### Protein A purification and N-glycosylation analysis

The mAb in the culture supernatant was purified using a MabSelect SuRe Protein A column (GE Healthcare). The purified antibodies were subjected to N-glycosylation analysis using hydrophilic interaction chromatography (HILIC). The protocols for protein A purification and glycosylation analysis have been described in a previous study^29^. Additional information on the confirmation of the glycan structure identity is provided in the supplementary materials. The abundance of each structure was determined as the percentage of total peak area based on the method described in a previous study^40^. The level of non-fucosylation was calculated as the combined relative abundance of non-fucosylated species, including both complex and hybrid structures. Galactosylation was determined as the combined relative abundance of mono- and bi-sialylated species. The hybrid-bisecting level was the relative abundance of M5A1B.

### Analysis of integration sites, mRNA, and protein levels

For southern blot analysis, genomic DNA (gDNA) was extracted from dMCL cell pellets using the PureLink Genomic DNA Mini Kit (Thermo Fisher) according to the manufacturer’s protocols. 5 µg of gDNA was digested with either XbaI, MfeI or NdeI restriction enzyme (NEB). The Southern probe was designed to target the NEO coding region in the landing pad 2. A 700-bp hybridization probe was generated by PCR using primer set (forward primer 5′-CTGATTGAACAAGATGGATTG-3′, reverser primer 5′-TCAGAAGAACTCGTCAAGAAG-3′) using PCR DIG probe synthesis kit (Roche). The probes were labelled with digoxigennin (DIG), followed by removal of free nucleotides using NucleoSpin Gel and PCR Clean-up column (Macherey–Nagel). The hybridization was performed as described previously^41^. Finally, the membrane was exposed to X-ray film for visualization.

Total RNA was isolated from ten million cells collected from each fed-batch culture using the RNeasy® Mini Kit (Qiagen). The mRNA levels for MANII, cGNTIII and β-actin were analyzed by quantitative real-time PCR (qRT-PCR) using Luna Universal qPCR Master Mix (NEB) and the LightCycler 480 (Roche) as described previously^32^. The primer sequences used for mRNA analysis were listed in Supporting Table S1.

To carry out Western blotting for protein level analysis, ten million cells were first homogenized in the CelLytic M (Sigma). The concentration of proteins in the cell lysates was then quantified by Pierce BCA protein assay (Themo Fisher). Subsequently, 10 µg of proteins were fractionated on a 4–12% gradient PAGE gel (Thermo Fisher). The separated proteins in the gel were then transferred to a PVDF membrane using iBlot® (Thermo Fisher). The PVDF membrane was blocked for 1 h in 1× Tris buffered saline supplemented with 0.1% Tween20 (First Base, Singapore) and 5% non-fat milk. It was then probed with primary mouse anti-Myc antibody (1:500) (RnD), or GAPDH (1:1000) (Abcam) overnight at 4 °C. The membrane was subsequently washed with TBST and then incubated with 1:5000 corresponding diluted secondary antibody (Promega) in blocking buffer for 1 h at room temperature (RT). After another round of washing, the membrane was visualized using Amersham ECL system (GE Healthcare Life Sciences). The human MANII and chimeric cGNTIII enzymes were differentiated based on their respective molecular weights of 130 kDa and 70 kDa, respectively.

### Effector cell activation (ECA) assay

The levels of ECA induced by different antibody samples were determined by using a ADCC Reporter Bioassay kit (Promega) according to manufacturing protocol. Briefly, CD20 positive WIL2-S cells were thawed and seeded into white, flat-bottom 96-well assay plates (Corning) at the recommended density. A three-fold serial dilution of each glycoengineered Rituximab was added to the wells. Effector cells were thawed and added into each well at a concentration of 75,000 cells/well, resulting in a target: effector ratio of 1:6. The plate was then incubated for 6 h at 37 °C. Thereafter, Bio-Glo Luciferase Assay Reagent was added into each well and incubated for 10 min before being subjected to luminescence measurement using an Infinite M200 Pro plate reader (Tecan). The levels of ECA were quantified with the luminescence readout.

### Complement-dependent cytotoxicity (CDC) assays

Complement-dependent cytotoxicity (CDC) was determined by measuring the release of lactate dehydrogenase (LDH) after incubation with glycoengineered Rituximab in the presence of human complement serum using the CytoTox 96® Non-Radioactive Cytotoxicity Assay (Promega). Briefly, CD20 positive Raji cells were seeded into flat-bottom 96-well tissue culture plates (Corning) in RPMI media containing 4% human complement sera (Sigma). A four-fold serial dilution of each glycoengineered Rituximab was added and incubated for one hour at 37 °C. After incubation, the plates were centrifuged at 250 × g for 4 min, and the 50 µL of the supernatant transferred to black, flat-bottom 96-well assay plates (Corning). 50 µL of Substrate Mix was added and incubated in the dark for an additional 30 min. The reaction was stopped by the addition of Stop Solution, and the absorbance at 490 nm was measured using Infinite M200 Pro plate reader (Tecan). Background absorbance from the “Media-only” wells was subtracted from the absorbance values. The percentage of specific cell lysis was calculated according to the manufacturer's instructions using the following formula: 100 × [(A−C)/(B−C)], where A represents the absorbance obtained with antibody-complement (test release), B represents the absorbance obtained by lysing all target cells with 1% Triton X-100 (maximal release), and C represents the absorbance obtained with target cells with complement sera only (minimal release).

### Supplementary Information

Supplementary Information.

## Data Availability

All data generated or analysed during this study are included in this published article and its supplementary information files.
