# Supplementary material for: Optimizing effector functions of monoclonal antibodies via tailored N-glycan engineering using a dual landing pad CHO targeted integration platform
Source: Sci Rep. 2023 Sep 20;13:15620. doi: 10.1038/s41598-023-42925-1 (PMC10511539; doi:10.1038/s41598-023-42925-1)
Supplement: Supplementary file 1 — Supplementary Information. [file 41598_2023_42925_MOESM1_ESM.pdf]

Supplementary information

Title

Optimizing effector functions of monoclonal antibodies via tailored N-glycan engineering using a dual landing pad CHO targeted integration platform

Authors and affiliations

Ngan T.B. Nguyen, Hau Wan Leung, Kuin Tian Pang, Shi Jie Tay, Ian Walsh, Andre B.H. Choo, Yuan Sheng Yang*

Bioprocessing Technology Institute (BTI), Agency for Science, Technology and Research (A*STAR), Singapore, Singapore

***Corresponding author: Dr. Yuan Sheng Yang, Bioprocessing Technology Institute, Agency for Science, Technology and Research (A*STAR), 20 Biopolis Way, #06-01 Centros, Singapore 138668 (yang_yuansheng@bti.a-star.edu.sg)

Supplementary table 1. List of primers for RT-PCR analysis.

	Gene	Forward primer (5'-3')	Reverse primer (5'-3')
1	**MANII**	ACTCAGAGAGACAAGGGCCAATC	TCTGTCAGTGCCGTGTAAAGTGA
2	**cGNTIII**	CCGGAGGACACCACAGAGTATTT	CACCTTGGTCTTCTCCTCTGTCC
3	**ACTIN**	AGCTGAGAGGGAAATTGTGCG	GCAACGGAACCGCTCATT

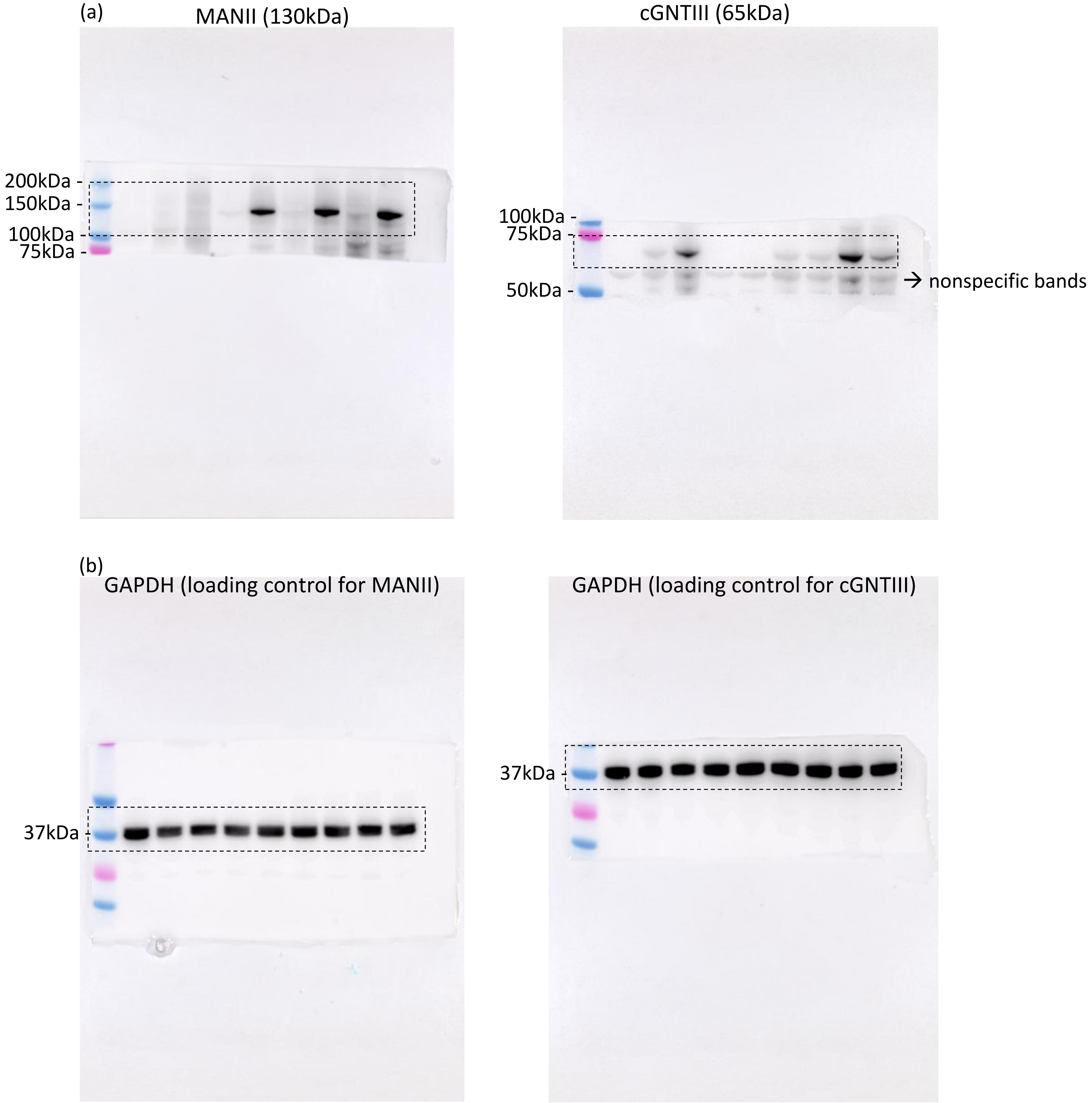

Supplementary Figure S1. Samples are separated on two SDS-PAGE gels. Separated proteins in each gel were then transferred onto PVDF blotting membranes. Each membrane was then cut in two halves at 75kDa and 50kDa marker bands and immunoblotted with the respective antibodies. The upper halves were used to detected MANII and cGNTIII proteins, while the corresponding halves were used to detected loading control (GAPDH). (a) Uncropped image for figure 3C (ManII and cGNTIII panels). (b) Uncropped image for figure 3C (GAPDH panels). The dotted lines indicated where the images were cropped from the original blots and presented in the main figure 3C. The images' brightness and contrast were not adjusted, though the images were recoloured into grayscale and enlarged.

Confirmation of N-glycan structure identify

We utilized data from the M_h+G_h sample as an illustrative example to demonstrate the process of determining the identity of each glycan structure. The same methodology was applied to the other samples. Hydrophilic interaction chromatography (HILIC)-Fluorescence-MS was employed for the separation of fluorescently labeled N-glycans, and parallel runs of external standards were conducted to facilitate retention time separation. The data obtained from HILIC-FLR was processed and analyzed using the UNIFI Biopharmaceutical software platform (version 1.8). The retention time of each chromatographic peak was converted into glucose units (GU) using a calibration curve established with an external standard of RapiFluor-MS labeled dextran glucose homopolymer ladder (Waters Corporation), utilizing a cubic spline fit[1]. This approach ensured consistent GU values despite variations in retention time, enabling the matching of GU values with a database in UNIFI. The summarized table below presents the identified glycans in M_h + G_h, confirming that all GU values were within a range of +/-0.3 units.

Supplementary table 2. Summary table of each of the glycan structures found in M_h + G_h. * indicates isomer.

Glycan	Observed RT (min)	Expected GU	Observed GU	Observed m/z	Charge	% Amount (%)	Fragments detected
A2	10.24	5.46	5.65	814.84	2	1.98	-
F(6)A2	11.14	5.79	5.98	887.86	2	9.75	-
F(6)A2B	12.16	6.12	6.35	989.41	2	2.04	-
A2[6]G(4)1	12.43	6.18	6.44	895.86	2	10.18	-
F(6)A2[6]G(4)1	13.35	6.53	6.78	968.89	2	3.87	-
F(6)A2[3]G(4)1	13.73	6.66	6.92	968.89	2	1.37	-
M5A1B	15.08	-	7.41	976.89	2	34.57	Yes
F(6)A2G(4)2	15.87	7.43	7.70	1049.92	2	4.56	-
M5A1BG(4)1	17.07	-	8.15	1057.92	2	2.81	Yes
M5A1BG(4)1*	17.37	-	8.26	1057.92	2	6.14	Yes
M5A1BG(4)1 S(3)1	18.76	-	8.81	1203.46	2	2.88	Yes

In our N-glycan identification, we enhanced sensitivity significantly by combining MS with HILIC-UPLC detection. This enabled us to determine both the mass of the glycans and the GU values in the HILIC layer. This UPLC-MS approach has previously demonstrated successful N-glycan detection in rituximab (2) and another approach where retention time and mass matching were utilized similar to this work (3). The observed mass of each glycan is presented in Table 2, with all glycan masses confirmed within 10 PPM.

Furthermore, the mass of glycan fragments can serve as confirmation of the structure. Hybrid structures were confirmed by a diagnostic ion with a mass of 1101.48, corresponding to a bisecting structure fragment (Supplementary Figure 2A), while a mass of 773.81 corresponding to an M5 fragment was identified (Supplementary Figure 2A). This information is indicated in Table 2, where we specify that the fragments were detected for these structures.

Finally, human serum IgG was run in the same batch as the samples. Supplementary Figure 2B displays stacked chromatograms of the Mh + Gh sample and Human serum IgG. It is noteworthy that the GU values and observed mass for two of the bisecting structures, F(6)A2BG(4)1 and F(6)A2BG(4)1, match in both the Mh + Gh sample and Human serum IgG (Supplementary Figure 2B). This confirmation allows us to establish the identity of these two structures.

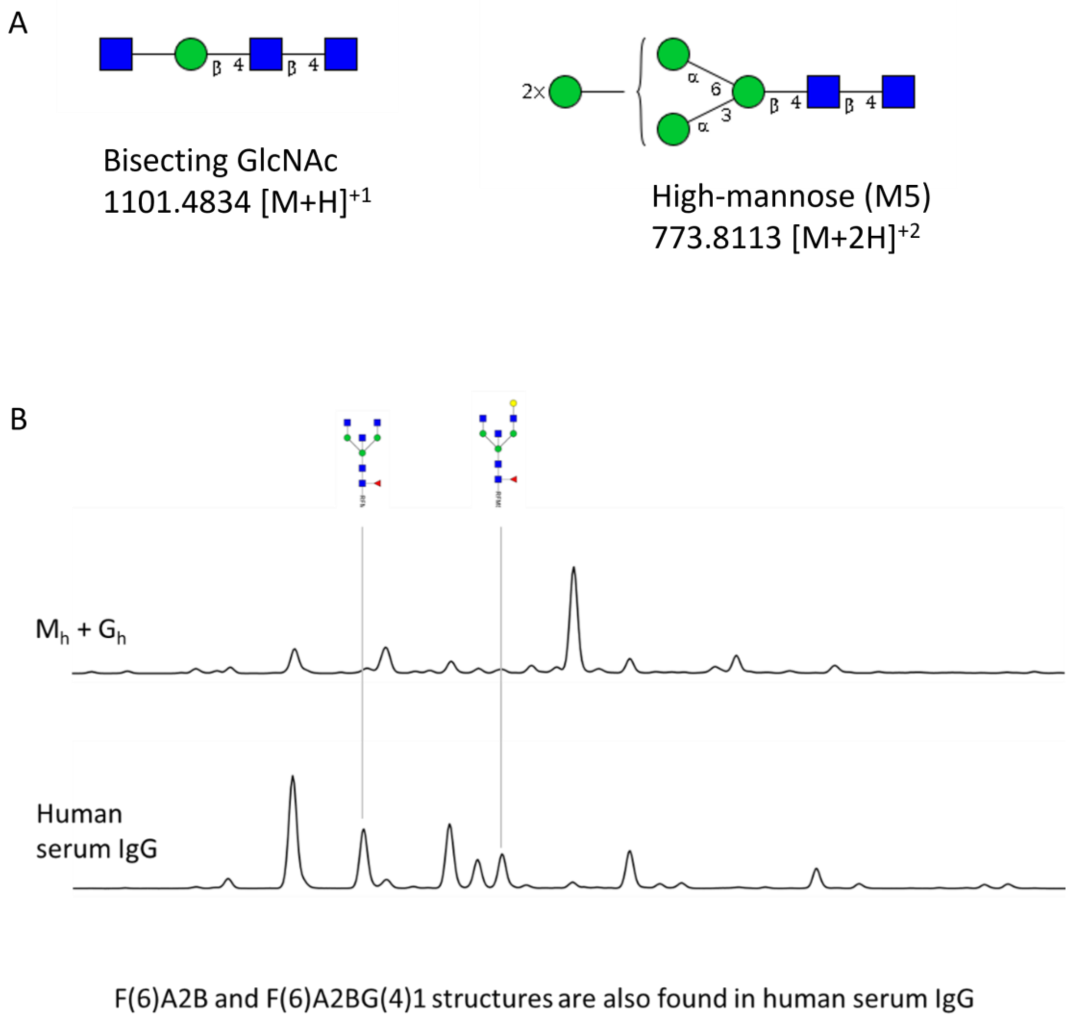

Supplementary Figure S2. (A) Bisecting and Mannose fragments identified for further confirmation (B) Human serum IgG overlay of chromatograms to identify bisecting fucosylated N-glycans.

Supplementary Table 3. Structure of complex and hybrid N-glycans for the summarize Oxford notation text format in Supplementary Table 2. The corresponding Symbol Nomenclature for Glycans (SNFG) for each oxford text format is SNFG is described in detail in the previous study[4].

Complex N-glycans

Oxford notation	Symbol Nomenclature for Glycans
A2	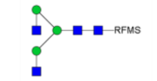
F(6)A2	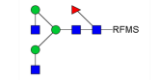
F(6)A2B	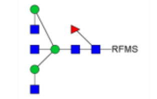
A2[6]G(4)1	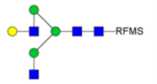
F(6)A2[6]G(4)1	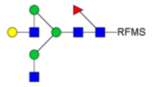
F(6)A2[3]G(4)1	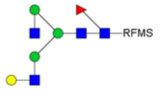
F(6)A2G(4)2	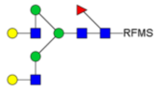

Hybrid N-glycans

Oxford notation	Symbol Nomenclature for Glycans
M5A1B	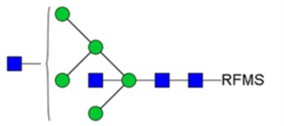
M5A1BG(4)1	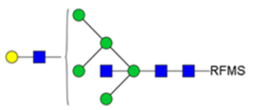
M5A1BG(4)1S(3)1	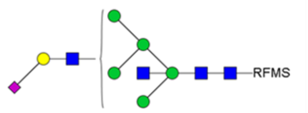

References:

1. Marino, Karina, et al. "A systematic approach to protein glycosylation analysis: a path through the maze." *Nature chemical biology* 6.10 (2010): 713-723.
2. Shang, Tanya Q., et al. "Development and application of a robust N-glycan profiling method for heightened characterization of monoclonal antibodies and related glycoproteins." *Journal of pharmaceutical sciences* 103.7 (2014): 1967-1978.
3. Song, Ting, et al. "In-depth method for the characterization of glycosylation in manufactured recombinant monoclonal antibody drugs." *Analytical chemistry* 86.12 (2014): 5661-5666.
4. Neelamegham, Sriram, et al. "Updates to the symbol nomenclature for glycans guidelines." Glycobiology 29.9 (2019): 620-624.
